# Toxic Epidermal Necrolysis

**Published:** 2014-03-15

**Authors:** Saami Khalifian, Zuhaib Ibrahim, Mohammed T. Lilo, Stephen M. Milner

**Affiliations:** ^a^Department of Plastic and Reconstructive Surgery, Johns Hopkins University School of Medicine, Baltimore, Md; ^b^Department of Pathology, Johns Hopkins University School of Medicine, Baltimore, Md

**Keywords:** toxic epidermal necrolysis, Lyell's syndrome, stevens-johnson syndrome, TEN, drug reaction

## DESCRIPTION

**Figure 1:**
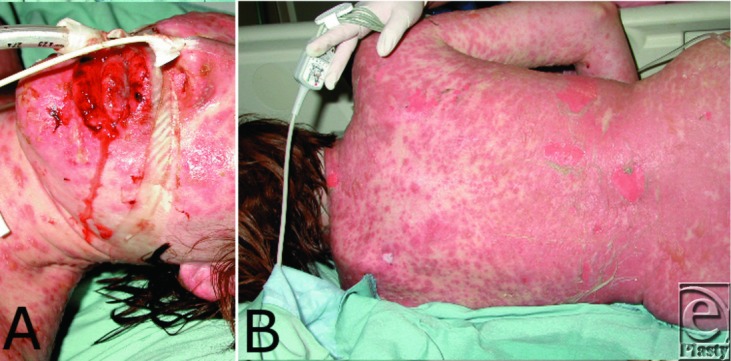
Generalized epidermolysis on anterior and posterior torso with crusty erosions on oral mucosa.

A 33-year-old woman presents with diffuse rash 3 weeks after completing a course of Bactrim for a urinary tract infection. Erythematous maculopapular rash is evident on the trunk and extremities with painful, epidermal sloughing on the back leaving a moist, denuded dermis. Note the hemorrhagic, crusty erosions on the oral mucosa (Fig 1).

## QUESTIONS

1. **What is the diagnosis?**
2. **What is the differential diagnosis?**
3. **What treatment options are available to patients with toxic epidermal necrolysis (TEN)?**
4. **Are particular prognostic factors associated with a higher risk of mortality?**

## DISCUSSION

**Figure 2:**
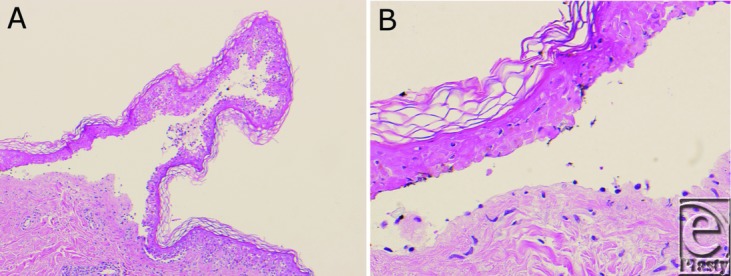
Characteristic histopathologic changes in toxic epidermal necrolysis. (a) There is a broad, paucicellular, subepidermal blister with confluent, full-thickness necrosis of the blister roof. At the edge of the blister, there is prominent dyskeratosis and vacuolar alteration of the epidermal basal layer (hematoxylin and eosin, original magnification 100X). (b) There is an unaltered, basket weave stratum corneum and a relative absence of inflammation (hematoxylin and eosin, original magnification 400X).

The patient has TEN characterized by destruction and detachment of skin epithelium and mucous membranes, most often precipitated by medications. It typically presents with an influenza-like prodrome of abrupt onset, including high fever, systemic toxicity, nausea or vomiting, conjunctivitis, and pharyngitis lasting days to weeks.^1^ Lateral pressure on the epidermis often causes detachment from the dermis (positive Nikolsky sign). The rash typically occurs on the trunk, extremities, and face, but spares the scalp.^2^ Hemorrhagic, crusty erosions on the oral mucosa and ocular pseudomembrane formation of the eyes are also common. Medications are the major precipitating cause, although infectious and idiopathic etiologies are possible.^3^ Toxic epidermal necrolysis is a clinical diagnosis with no definitive laboratory test. Histological analysis of affected skin shows full-thickness epithelial necrosis and detachment with necrotic keratinocytes in the epidermis (Fig 2).^4^ The density of the dermal infiltrate correlates with the severity of the disease. Leukopenia and anemia are common; however, TEN affects multiple organ systems as it progresses, which causes a variety of laboratory abnormalities.

The differential diagnosis includes staphylococcal scalded skin syndrome, pemphigoid and pemphigus, paraneoplastic pemphigus, linear IgA dermatosis, acute graft-versus-host disease, and acute generalized exanthematous pustulosis. Toxic epidermal necrolysis can be differentiated from these by the presence of fever, mucositis, acute onset with rapid course, and morphology of cutaneous manifestations.^1^ Mucositis (commonly of the oropharynx, eyes, or genitalia) in TEN typically precedes the cutaneous eruption, which begins as a warm, dusky, erythematous, maculopapular rash, followed by painful blistering and epidermal sheet detachment leaving a denuded dermis.

Early discontinuation of offending agents and admission to a burn unit with intensive supportive care are directly related to survival.^5^ Sepsis is a major complication; therefore, blood cultures are recommended with initiation of directed antibiotic therapy upon early signs of sepsis.^6^ Overt necrotic epidermis can be debrided and should be covered with antibacterial dressings. Frequent administration of eye lubrication and lysis of ocular adhesions is sometimes necessary.^5^ No specific treatment has been proven to be effective in a randomized controlled trial; however, some studies suggest that plasmapheresis, tumor necrosis factor-α inhibitors, or intravenous immunoglobulin may improve outcomes.^1^

The SCORTEN^7^ level is used to predict disease severity and mortality, and is calculated within 24 hours of admission and on day 3. The score is the sum of 7 variables, each contributing 1 point, including the following:

1. Age > 40 years
2. Heart rate > 120 beats per minute
3. Positive cancer history
4. Epidermal detachment > 10% body area on day 1
5. Blood urea nitrogen > 28 mg/dL
6. Glucose > 252 mg/dL
7. Bicarbonate < 20 mEq/L

A score of 2 or less portends 12% mortality or less, while a score of 4 predicts 58% mortality, and 5 or more is associated with 90% death rate. Leukopenia, thrombocytopenia, delay in hospital admission, and prehospital treatment with corticosteroids or antibiotics are also poor prognostic factors.^1^

In summary, TEN is an acute, severe dermatological disease characterized by destruction and detachment of skin epithelium and mucous membranes, most often precipitated by medications. Initial manifestations are nonspecific; however, the rash rapidly progresses to become painful, vesicular, and bullous. Treatment is supportive and includes withdrawal of offending drug and admission to a burn unit. The SCORTEN scale accurately predicts mortality.
